# Pre-treatment Amino Acids and Risk of Paclitaxel-induced Peripheral Neuropathy in SWOG S0221

**DOI:** 10.21203/rs.3.rs-3242513/v1

**Published:** 2023-09-01

**Authors:** Ciao-Sin Chen, Gary Zirpoli, G. Thomas Budd, William E. Barlow, Lajos Pusztai, Gabriel N. Hortobagyi, Kathy S. Albain, Andrew K. Godwin, Alastair Thompson, N. Lynn Henry, Christine B. Ambrosone, Kathleen A. Stringer, Daniel L Hertz

**Affiliations:** University of Michigan College of Pharmacy; Boston University Slone Epidemiology Center; Cleveland Clinic; SWOG; Yale University School of Medicine; The University of Texas MD Anderson Cancer Center; Loyola University Medical Center; University of Kansas Medical Center; Baylor College of Medicine; University of Michigan Rogel Cancer Center; Roswell Park Comprehensive Cancer Center; University of Michigan College of Pharmacy; University of Michigan College of Pharmacy

**Keywords:** Chemotherapy-induced peripheral neuropathy, paclitaxel, histidine, amino acid, pharmacometabolomics, predictive biomarkers, risk factors, symptom science

## Abstract

**Background:**

Chemotherapy-induced peripheral neuropathy (CIPN) is a treatment-limiting and debilitating neurotoxicity of many commonly used anti-cancer agents, including paclitaxel. The objective of this study was to confirm the previously found inverse association between pre-treatment blood concentrations of histidine and CIPN occurrence and examine relationships of other amino acids with CIPN severity.

**Methods:**

Pre-treatment levels of 20 amino acid concentrations were measured via a targeted mass spectrometry assay in banked serum from the SWOG S0221 (NCT00070564) trial of patients with early-stage breast cancer receiving paclitaxel. The associations between amino acid levels and CIPN occurrence or severity were tested in regression analysis adjusted for paclitaxel schedule, age, self-reported race, and body mass index with Bonferroni correction for multiple comparisons. The network of metabolic pathways of amino acids was analyzed using over-representation analysis in MetaboAnalyst. The partial correlation network of amino acids was evaluated using a debiased sparse partial correlation algorithm and Cytoscape.

**Results:**

In the primary analysis, histidine concentration was not associated with CIPN occurrence (odds ratio (OR) = 0.97 [0.83, 1.13], p = 0.72). In a secondary analysis, no amino acid was associated with CIPN occurrence (all p > 0.0025). Higher concentrations of four amino acids, glutamate (β = 0.58 [0.23, 0.93], p = 0.001), phenylalanine (β = 0.54 [0.19, 0.89], p = 0.002), tyrosine (β = 0.57 [0.23, 0.91], p = 0.001), and valine (β = 0.58 [0.24, 0.92], p = 0.001) were associated with more severe CIPN, but none of these associations retained significance after adjustment. In the over-representation analysis, no amino acid metabolic pathways were significantly enriched (all FDR > 0.05). In the network of enriched pathways, glutamate metabolism had the highest centrality.

**Conclusions:**

This analysis showed that pre-treatment serum amino acid concentrations are not strongly predictive of CIPN severity. Future prospectively designed studies that assess non-amino acid metabolomics predictors are encouraged.

## Introduction

Chemotherapy-induced peripheral neuropathy (CIPN) is a treatment-limiting neurotoxicity of many commonly used anti-cancer agents, including paclitaxel. Up to 70% of patients receiving paclitaxel experience CIPN[1] and approximately 30% experience severe symptoms.[2] CIPN can last for years after finishing chemotherapy,[3, 4] which significantly diminishes patients’ long-term quality of life.[5] Duloxetine is the only drug that has demonstrated efficacy in CIPN pain management.[6] There are no established strategies to prevent sensory or motor CIPN symptoms.[7] Therefore, neurotoxic chemotherapy treatment is often delayed, dosing is reduced, or treatment is discontinued early in patients with moderate or severe CIPN, which diminishes drug efficacy and cancer survival.[8, 9] It is critical to discover predictors of CIPN that can be used to develop approaches to prevent or treat CIPN and improve treatment outcomes in patients with cancer.

A previous analysis conducted within an observational study ancillary to the prospective phase III SWOG S0221 clinical trial[10] found that patients who reported greater dietary intake of grain and less intake of citrus fruits had greater risk of peripheral neuropathy[11]. These results suggest that there may be some nutritional signatures that can be used to predict which patients will experience CIPN[12]. Metabolomics is a field of biomarker science that measures small molecules within biological samples, and has been used to predict CIPN in pediatric patients receiving vincristine.[13] In our previous metabolomics study of 48 patients with breast cancer, whole blood levels of histidine prior to start of weekly paclitaxel treatment were inversely associated with CIPN severity.[12] The objective of this study was to confirm the previously detected association between pre-treatment histidine and CIPN occurrence and assess the association of other amino acids with CIPN severity in a large cohort of patients with breast cancer receiving paclitaxel treatment in S0221.

## Methods

### Study patients and clinical data

The clinical data and sample collection for this analysis have been previously described.[10, 14] Briefly, this secondary biomarker analysis was conducted using data and serum samples collected within SWOG S0221 (NCT00070564). S0221 was a phase III clinical trial that randomized patients with early-stage breast cancer to two schedules of doxorubicin/cyclophosphamide, followed by paclitaxel 80 mg/m^2^ QW for 12 doses vs. 175mg/m^2^ Q2W for 6 doses. Pre-existing neuropathy was not an exclusion criterion and was not documented. Adverse events were assessed every 4 weeks using the National Cancer Institute Common Terminology Criteria for Adverse Events (NCI CTCAE) version 3.0.[15] The primary endpoint for our analysis was grade 3 or higher (grade 3+) sensory CIPN that was possibly, probably, or definitely related to chemotherapy treatment. Patient characteristics were compared between patients with and without grade 3 + sensory CIPN using a t-test for continuous variables and a chi-squared test for categorical variables.

**Figure 1:**
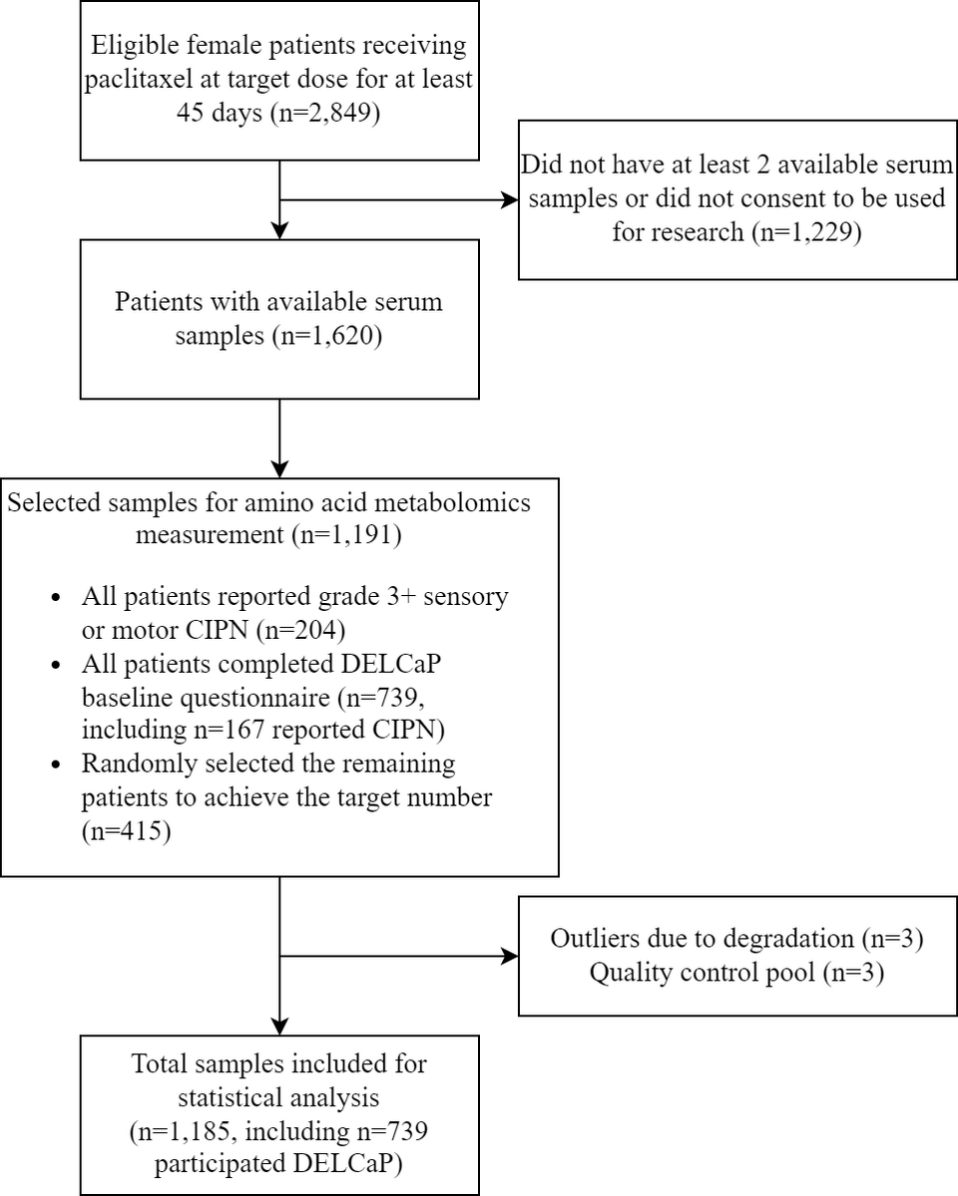
Sample selection of amino acid analysis. Of the 2,849 eligible female participants receiving paclitaxel in S0221, 1,620 were eligible for this biomarker study. A total of 1,191 patient samples were selected for amino acid metabolomics analysis, and 1,185 of these were included in the primary analysis. A subset of patients (n=739) also participated in the DELCaP substudy and completed the patient-reported CIPN questionnaire at the end of treatment.

From the 2,849 female participants who received paclitaxel in S0221, 1,191 who had at least two baseline blood specimens available and consented to use of their samples in future research were selected through a process that has been described in detail[16] [ASCO ABSTRACT REFERENCE TO BE REPLACED BY JNCCN ARTICLE REF (IN PRESS) DURING REVISION] (Fig. 1). Briefly, participants who reported grade 3 + sensory or motor CIPN (n = 204) or completed the DELCaP substudy[17] questionnaires (additional n = 572) were prioritized, followed by random selection from the remaining eligible participants. The DELCaP questionnaire collected patient-reported outcomes via the Functional Assessment of Cancer Therapy (FACT)-taxane questionnaire before and after chemotherapy.[11, 14] A secondary CIPN endpoint was created using the sum score of the first 4 sensory items, including numbness or tingling in the hands, numbness or tingling in the feet, discomfort in the hands, and discomfort in the feet.[18, 19]

### Targeted quantitative amino acid assay

Blood samples were collected and processed at enrollment; the resulting serum was stored (−80°C) until the time of assay. Samples were randomized into 15 batches and levels of 20 amino acids were quantified in a blinded manner using EZ:faast (Phenomenex, Torrance, CA), a targeted electron ionization gas chromatography-mass spectrometry (EI-GC/MS) assay through the Michigan Regional Comprehensive Metabolomics Resource Core (MRC2) (Ann Arbor, MI).[20] A quality control pool of 3 samples was used for data processing and these samples were not included in the statistical analysis (Fig. 1). Before statistical analysis, batch effects were corrected by systematic error removal using random forest (SERRF).[21] Outliers were examined using principal component analysis, and three outlier samples were removed because of higher-than-usual concentration profiles due to suspected sample degradation (**Supplemental Fig. 1**). The concentration measurements for one sample with a volume less than 100 μL (80 μL) were adjusted proportionally. Amino acid concentration data were log_2_ transformed to meet the assumption of normal distribution.

### Regression analysis

The analysis was conducted following an *a priori* plan agreed upon by the study team and the SWOG Statistical Data Management Center. The data were further z-score normalized based on the mean and standard deviation of each amino acid. Logistic regression was used to assess the relationship between grade 3 + sensory CIPN and pre-treatment concentrations of each amino acid. Bonferroni correction was used in each regression analysis for multiple comparisons (α = 0.05/20 = 0.0025). The *a priori* primary analysis attempted to validate the inverse association of histidine with CIPN;[22] all the other analyses were conducted as secondary. Any association detected in univariate analysis was then tested in the entire cohort and within each paclitaxel treatment arm (QW or Q2W) adjusted for the following covariates that have been reported to be associated with CIPN risk: age, self-reported race (white vs. Black vs. other), and body mass index (BMI) via multivariable logistic regression. Information on diabetes and other metabolic disorders was not collected on S0221 and was not available to be included in the analysis. Among the patients who participated in the DELCaP substudy, linear regression was conducted, similar to the primary analysis, using end-of-treatment FACT4 as the dependent variable with adjustment for baseline FACT4. All regression analyses were conducted using R 4.2.1.[23]. Missing values of baseline FACT4 were imputed using functions mice and pool in mice package with 100 iterations to average from 5 imputed datasets.[24]

### Pathway enrichment and network analysis

Metabolic pathways of amino acids with a p < 0.05 in any regression analyses were assessed in over-representation analysis using a metabolomics webtool, MetaboAnalyst,[25] with Small Molecule Pathway database.[26] The enrichment ratio was calculated as the observed counts of metabolites divided by the expected counts. Benjamini and Hochberg false discovery rate (FDR) was reported. The network of enriched pathways was generated by MetaboAnalyst,[25] in which pathways were connected if the number of shared metabolites was > 25% of the combined sets. The partial correlation network of amino acids with a p < 0.05 in any regression analyses was built using debiased sparse partial correlation algorithm[27] and MetScape[28] in Cytoscape.[29] The edges were selected if FDR < 0.05. Degree centrality, betweenness centrality, and stress centrality were calculated using NetworkAnalyzer[30] in Cytoscape.[29]

## Results

### Study patients and amino acids concentration

Among the 1,185 participants included in the analysis (Figure 1), the mean age was 51 years (SD=10) and 84% were self-reported white. Table 1 shows characteristics of patients by whether they developed grade 3+ sensory CIPN (16%) or not. In the principal component analysis of amino acid metabolomics data, excluding the three outlier samples, the batch-corrected variation was nominal but there was no separation between patients who did and did not develop grade 3+ sensory CIPN (**Supplemental Figure 1**).

### Amino acids associated with sensory peripheral neuropathy

**Figure 2:**
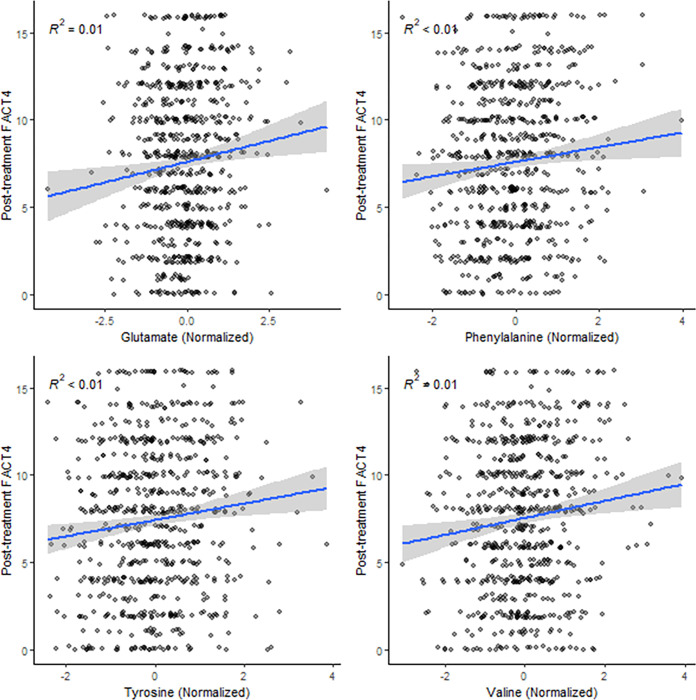
Amino acid concentrations associated with patient-reported peripheral neuropathy severity. Higher concentrations of four amino acids, glutamate, phenylalanine, tyrosine, and valine were associated with more severe patient-reported CIPN. Each dot represents a patient. The regression line between amino acid levels and post-treatment FACT4 score was annotated with confidence intervals.

In the primary analysis, histidine concentration was not associated with grade 3+ CIPN (univariate regression: OR=0.97 [0.83, 1.13], p=0.72, Table 2). In a secondary analysis, no amino acid was associated with the incidence of grade 3+ sensory CIPN (all p>0.0025, Table 2). In secondary analyses of patient-reported CIPN severity, higher concentrations of 4 amino acids, glutamate (β=0.58 [0.23, 0.93], p=0.001), phenylalanine (β=0.54 [0.19, 0.89], p=0.002), tyrosine (β=0.57 [0.23, 0.91], p=0.001), and valine (β=0.58 [0.24, 0.92], p=0.001) were associated with higher post-treatment FACT4, when adjusted for baseline FACT4 (Figure 2). However, none of these associations retained significance in the entire cohort or either treatment arm after adjusting for additional clinical covariates (all p>0.0025, Table 2).

### Pathway enrichment and network analysis

**Figure 3:**
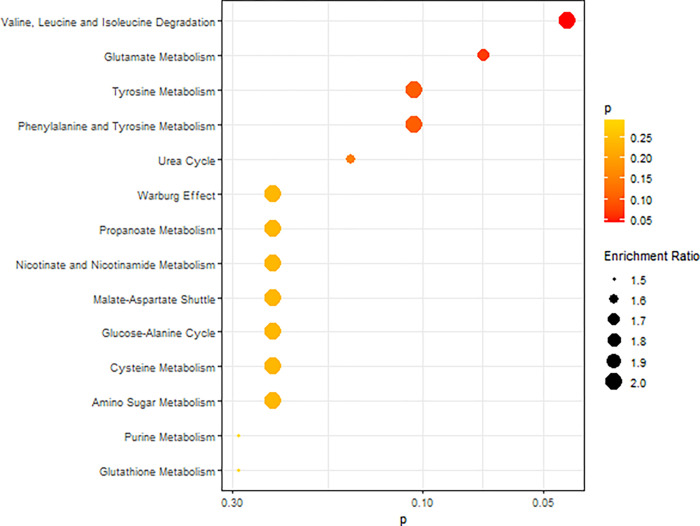
Top enriched pathways of amino acid-related metabolism. The over-representation analysis identified 14 pathways with FDR<50%. The color scale darkens with decreasing p value. The dot size represents the enrichment ratio, which is the observed counts divided by the expected counts.

**Figure 4:**
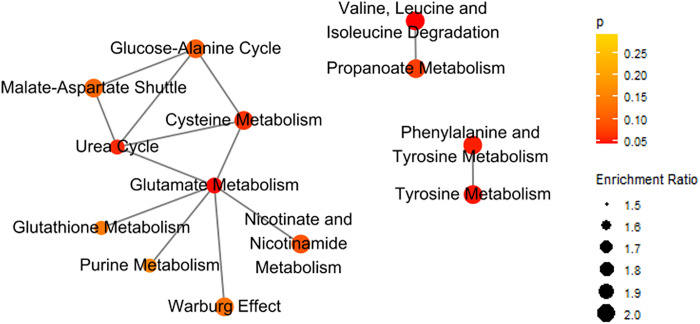
Network of enriched metabolic pathways of amino acids associated with peripheral neuropathy. Pathways were marked as connected if the number of shared metabolites was >25% of the combined sets. Glutamate metabolism had the highest degree centrality and betweenness centrality. The color scale darkens with decreasing p value. Dot size is correlated with enrichment ratio.

In the over-representation analysis, no amino acid metabolic pathways were significantly enriched (all FDR>0.05). Among 14 metabolic pathways with FDR<50%, the most strongly associated pathways are valine, leucine, and isoleucine degradation (p=0.043) and glutamate metabolism (p=0.070). (Figure 3 and **Supplemental Table 1**). In the network of enriched pathways, glutamate metabolism had the highest centrality (degree=6, betweenness=0.80, stress=52, Figure 4 and **Supplemental Table 2**).

## Discussion

CIPN is a debilitating neurotoxicity of paclitaxel that limits the treatment efficacy and reduces quality of life. The primary objective of this analysis was to validate the inverse association of CIPN occurrence with pre-treatment histidine;[22] however, no association was identified. In a secondary analysis of other amino acids, higher glutamate, phenylalanine, tyrosine, and valine were weakly associated with higher CIPN severity. Metabolic network analyses suggested that associations of amino acids with CIPN might act through a metabolic pathway mediated by glutamate.

Our prior analysis of a smaller patient cohort found an inverse relationship between whole blood levels of histidine, phenylalanine, and threonine and CIPN severity.[12] In this analysis of the much larger S0221 clinical trial cohort using archival serum samples, our previous findings were not reproduced. However, unexpectedly, phenylalanine, tyrosine, glutamate, and valine were associated with CIPN severity. Likely explanations for the different findings between the two studies may be the sample type (whole blood vs. serum)[31–33], the timing of blood collection (before paclitaxel vs. before any chemotherapy), and/or the duration the samples have been stored.

Phenylalanine is an essential aromatic amino acid supplied from dietary protein sources and is the precursor of tyrosine. Tyrosine is rapidly metabolized to 3,4-dihydroxyphenylalanine (levodopa), which is the precursor of many catecholamine neurotransmitters in the brain and is associated with many neurological diseases.[34] Prior research in humans and animals showed that genetic diseases that lead to the accumulation of phenylalanine can cause impairment of cognitive development, which requires the restriction of dietary phenylalanine intake. The resulting tyrosine deficiency has been suggested to be the cause of sensory neuropathy symptoms,[35] but a meta-analysis showed that tyrosine supplementation did not improve neuropsychological performance.[36] Whereas the current analysis found that patients with higher glutamate had more severe CIPN, several clinical trials have tested supplementation of glutamate or glutamine to reduce the severity of CIPN from paclitaxel, oxaliplatin, and vincristine, but the results were inconclusive.[37–43] Higher plasma glutamate has been reported in patients with more severe chronic pain syndromes caused by trauma,[44] but we are not aware of any studies comparing glutamate levels in patients with varying severity of CIPN.

A previous analysis conducted patients receiving vincristine suggested that machine learning approaches could be used to identify metabolomic signatures prior to and during treatment that were indicative of vincristine-induced peripheral neuropathy.[13] The investigators provide tools that could be used to predict a patient’s likelihood of CIPN, presumably to inform treatment decisions. We considered predictive CIPN models in the S0221 cohort using various machine learning techniques, however, these models had poor predictive performance and were therefore not included in this report. Our inability to validate our previously discovered amino acid biomarker candidates, or to identify sufficiently predictive amino acid signatures, demonstrates the importance of conducting statistically robust biomarker research prior to recommending biomarkers be used for clinical decision making. Although we find limited evidence supporting the use of amino acid metabolomics to predict CIPN, the amino acid signature in this analysis may provide intriguing avenues for further exploration of the biological mechanism underlying CIPN.

Although this is the largest CIPN metabolomics analysis that has been conducted to date and used CIPN data prospectively collected within a well-conducted clinical trial, there are several limitations of this analysis that should be considered. S0221 only collected CTCAE grade 3 + adverse events, which could have missed patients whose paclitaxel treatment was modified before they reached this level of severity, especially since dose adjustment data were not collected. Additionally, S0221 did not exclude patients with pre-existing neuropathy, who are particularly sensitive to peripheral neuropathy that may be unrelated to amino acid deficiencies. Information on diabetes and hyperlipidemia was also not collected, so possible confounding effects of relevant disease states or recent diet could not be accounted for in our analysis of non-fasting pre-treatment samples[45, 46]. Finally, our metabolomics panel only included the 20 amino acids that comprise proteins. Further research is warranted using broader metabolomics panels and other omics approaches such as lipidomics, proteomics, and microbiomics to better understand the key features that predict the occurrence and severity of CIPN.

In conclusion, this analysis found minimal evidence that pre-treatment levels of amino acids, including histidine, are associated with CIPN from paclitaxel. The metabolic network suggested that the weak association between amino acids and CIPN might be through the metabolism of glutamate. Additional studies that assess non-amino acid metabolomics and other potential predictive biomarkers are encouraged to identify predictive biomarkers that can be used to inform patient care and/or improve mechanistic understanding of CIPN that can be used to develop effective treatment approaches that could long-term treatment outcomes in patients with cancer.

## Figures and Tables

**Table 1.** Characteristics of patients included in the amino acid analyses.

	All patients	No sensory CIPN	Sensory CIPN	p-value
	(n=1,185)	(n=991)	(n=194)	

Age (years)	51	51	53	**0.003**
Mean	10	10	10	
Standard deviation	44–59	44–58	46–60	
Interquartile Range				

Self-reported race	992 (84%)	850 (86%)	142 (73%)	**<0.001**
White	108 (9%)	76 (8%)	32 (16%)	
Black	85 (7%)	65 (7%)	20 (10%)	
Other or unknown				

Body mass index (kg/m^2^)	30	30	31	0.108
Mean	6	8	7	
Standard deviation	25–34	25–34	26–36	
Interquartile Range				

Paclitaxel Treatment Arm	609 (51 %)	544 (55%)	65 (34%)	**<0.001**
Weekly	576 (49%)	447 (45%)	129 (66%)	
Every other week				

% of planned paclitaxel dose administered	88	91	70	**<0.001**
Mean	22	20	23	
Standard deviation	83–100	92–100	50–83	
Interquartile Range				

Motor CIPN Grade 3+	21 (2%)	8 (1 %)	13 (7%)	**<0.001**

Vitamin D insufficiency (≤20 ng/mL)	396 (33%)	314 (32%)	82 (42%)	**0.006**

Data are presented as N (%) or mean, standard error, and interquartile range. p-values are from comparing patients with and without grade 3+ sensory CIPN using a t-test for continuous variables and a chi-squared test for categorical variables. ER, estrogen receptor; HER2, human epidermal growth factor receptor 2; PgR, progesterone receptor.

**Table 2.** Regression analyses between amino acid concentrations and the occurrence or severity of sensory peripheral neuropathy.

	Occurrence of grade 3+ sensory CIPN				Post-treatment FACT4 scores as the severity of sensory CIPN			
	Without adjustment		Adjustment for paclitaxel arm, age, race, BMI		Adjustment for baseline FACT4		Adjustment for baseline FACT4, paclitaxel arm, age, race, BMI	
	OR [95% CI]	p-value	Adjusted OR [95% CI]	p-value	β [95% CI]	p-value	Adjusted β [95% CI]	p-value
Histidine^a^	0.97 [0.83, 1.13]	0.722	1.01 [0.86, 1.18]	0.878	−0.06 [−0.41, 0.29]	0.735	0.10 [−0.24, 0.44]	0.567
Alanine	1.22 [1.05, 1.44]	0.013	1.21 [1.02, 1.44]	0.026	0.40 [0.05, 0.74]	0.024	0.26 [−0.09, 0.60]	0.143
Arginine	1.01 [0.86, 1.17]	0.948	0.99 [0.85, 1.16]	0.882	0.24 [−0.11, 0.59]	0.176	0.16 [−0.22, 0.54]	0.411
Asparagine	0.94 [0.80, 1.10]	0.432	0.96 [0.82, 1.13]	0.643	−0.21 [−0.56, 0.14]	0.243	0.05 [−0.30, 0.39]	0.796
Aspartate	1.17 [1.00, 1.37]	0.047	2.54 [1.33, 5.04]	0.006	0.29 [−0.06, 0.64]	0.106	0.19 [−0.15, 0.53]	0.277
Cysteine	1.22 [1.04, 1.45]	0.018	1.13 [0.94, 1.36]	0.195	0.35 [0.01, 0.68]	0.042	0.09 [−0.26, 0.44]	0.599
Glutamate	1.14 [0.98, 1.33]	0.089	1.09 [0.92, 1.28]	0.333	0.58 [0.23, 0.93]	**0.001**	0.30 [−0.06, 0.65]	0.101
Glutamine	0.99 [0.85, 1.15]	0.886	2.79 [1.15, 6.97]	0.026	−0.01 [−0.37, 0.34]	0.939	0.08 [−0.27, 0.43]	0.661
Glycine	0.97 [0.83, 1.13]	0.714	1.05 [0.89, 1.24]	0.547	−0.21 [−0.55, 0.13]	0.227	0.06 [−0.28, 0.40]	0.728
Isoleucine	1.02 [0.87, 1.19]	0.829	0.98 [0.84, 1.16]	0.846	0.38 [0.04, 0.71]	0.030	0.29 [−0.04, 0.62]	0.088
Leucine	1.04 [0.90, 1.22]	0.575	1.00 [0.85, 1.18]	0.985	0.43 [0.09, 0.76]	0.014	0.33 [0.01, 0.66]	0.047
Lysine	0.95 [0.81, 1.11]	0.525	0.92 [0.78, 1.08]	0.298	0.30 [−0.04, 0.64]	0.087	0.20 [−0.14, 0.53]	0.253
Methionine	1.06 [0.91, 1.24]	0.425	1.04 [0.88, 1.21]	0.656	0.14 [−0.21, 0.48]	0.436	0.20 [−0.13, 0.53]	0.241
Phenylalanine	1.10 [0.94, 1.28]	0.240	1.04 [0.89, 1.22]	0.613	0.54 [0.19, 0.89]	**0.002**	0.42 [0.08, 0.76]	0.016
Proline	1.01 [0.87, 1.18]	0.854	1.01 [0.86, 1.19]	0.893	0.34 [0.00, 0.68]	0.051	0.29 [−0.04, 0.63]	0.084
Serine	0.94 [0.80, 1.09]	0.399	0.95 [0.81, 1.11]	0.494	−0.14 [−0.50, 0.21]	0.438	0.08 [−0.27, 0.43]	0.667
Threonine	1.04 [0.89, 1.21]	0.632	1.05 [0.89, 1.23]	0.585	0.16 [−0.18, 0.49]	0.365	0.30 [−0.03, 0.62]	0.077
Tryptophan	1.05 [0.90, 1.23]	0.514	1.09 [0.93, 1.28]	0.284	0.33 [−0.01, 0.67]	0.058	0.19 [−0.16, 0.55]	0.278
Tyrosine	1.18 [1.01, 1.37]	0.038	1.14 [0.97, 1.34]	0.115	0.57 [0.23, 0.91]	**0.001**	0.46 [0.13, 0.79]	0.007
Valine	1.11 [0.95, 1.29]	0.201	1.05 [0.89, 1.24]	0.556	0.58 [0.24, 0.92]	**0.001**	0.40 [0.07, 0.74]	0.020

a Pre-specified primary analysis was the association of histidine with grade 3+ sensory CIPN

OR, odds ratio; CI, confidence interval; β, regression coefficient; BMI, body mass index. Odds ratio and regression coefficients are presented as per standard deviation of individual amino acids. Bold indicates significant results after Bonferroni correction (p<0.0025).

## Data Availability

This data is available upon reasonable request to the corresponding author.
